# Use of a Regression Model to Study Host-Genomic Determinants of Phage Susceptibility in MRSA

**DOI:** 10.3390/antibiotics7010009

**Published:** 2018-01-29

**Authors:** Henrike Zschach, Mette V. Larsen, Henrik Hasman, Henrik Westh, Morten Nielsen, Ryszard Międzybrodzki, Ewa Jończyk-Matysiak, Beata Weber-Dąbrowska, Andrzej Górski

**Affiliations:** 1 Department of Bio and Health Informatics, Technical University of Denmark, 2800 Kgs. Lyngby, Denmark; 2 GoSeqIt ApS, Ved Klaedebo 9, 2970 Hoersholm, Denmark; MVL@goseqit.com; 3 Department of Bacteria, Fungi and Parasites, Statens Serum Institut, 2300 Copenhagen S, Denmark, henh@ssi.dk; 4 Department of Clinical Microbiology, MRSA Knowledge Center, Hvidovre Hospital, 2650 Hvidovre, Denmark; Henrik.torkil.westh@regionh.dk; 5 Faculty of Health and Medical Sciences, Institute of Clinical Medicine, University of Copenhagen, 2200 Copenhagen, Denmark; 6 Instituto de Investigaciones Biotecnológicas, Universidad Nacional de San Martín, San Martín, B 1650 HMP, Buenos Aires, Argentina; 7 Bacteriophage Laboratory, Hirszfeld Institute of Immunology and Experimental Therapy, Polish Academy of Sciences, 53-114 Wroclaw, Poland; mbrodzki@iitd.pan.wroc.pl (R.M.); ewa.jonczyk@iitd.pan.wroc.pl (E.J.-M.); weber@iitd.pan.wroc.pl (B.W.-D.); agorski@ikp.pl (A.G.); 8 Department of Clinical Immunology, Transplantation Institute, Medical University of Warsaw, 02-006 Warsaw, Poland

**Keywords:** phage therapy, bacterial phage resistance, regression modeling, MRSA

## Abstract

*Staphylococcus aureus* is a major agent of nosocomial infections. Especially in methicillin-resistant strains, conventional treatment options are limited and expensive, which has fueled a growing interest in phage therapy approaches. We have tested the susceptibility of 207 clinical *S. aureus* strains to 12 (nine monovalent) different therapeutic phage preparations and subsequently employed linear regression models to estimate the influence of individual host gene families on resistance to phages. Specifically, we used a two-step regression model setup with a preselection step based on gene family enrichment. We show that our models are robust and capture the data’s underlying signal by comparing their performance to that of models build on randomized data. In doing so, we have identified 167 gene families that govern phage resistance in our strain set and performed functional analysis on them. This revealed genes of possible prophage or mobile genetic element origin, along with genes involved in restriction-modification and transcription regulators, though the majority were genes of unknown function. This study is a step in the direction of understanding the intricate host-phage relationship in this important pathogen with the outlook to targeted phage therapy applications.

## 1. Introduction

Methicillin-resistant *Staphylococcus aureus* (MRSA) is a growing health concern. It is the agent of many chronic bacterial infections in hospitals as well as in the community. Its resistance to beta-lactamases severely limits treatment options, drives up the price for therapy, increases unwanted side effects, and leads in many cases to worse clinical outcomes [1]. MRSA has been classified as a high-priority pathogen on the 2017 list of antibiotic-resistant priority pathogens published by the World Health Organization [2]. Pathogens on this list are considered to pose the greatest threat to human health and to require urgently discovery and development of new antibiotics. 

Phage therapy has been proposed as a promising substitute for conventional antibiotics or a co-treatment in the treatment of multi-resistant bacterial pathogens [3,4,5,6,7]. Of the *S. aureus* phage known to date, most are temperate phages and belong to the Siphoviridae family [8]. Strictly lytic staphylococcal phages, as are typically required for therapy, are almost exclusively found in the Podoviridae and Myoviridae families [8].

The Hirszfeld Institute of Immunology and Experimental Therapy of the Polish Academy of Science in Wroclaw (HI) has been producing staphylococcal phages for therapeutic purposes since the 1970s [9]. At present, its collection consists of nine monovalent staphylococcal phages (see Materials and Methods) [10]. Those phages are used at the Phage Therapy Unit in Wrocław under the rules of a therapeutic experiment to conduct treatment of patients with chronic bacterial infections resistant to antibiotic therapy. The result have been encouraging, as a good response has been observed in one third of patients [6].

However, in order for phage therapy to be efficient, it is necessary to have a good understanding of the specific interaction between phage and host. There are many strategies by which bacteria aim to evade predation by phages, which is a significant fitness factor and therefore under high evolutionary pressure. *S. aureus* is known to be deficient in CRISPR, one of the major phage defense mechanisms [11]. Instead, its principle defense against invading DNAs are extensive restriction-modification (RM) systems [12]. RM systems are two-part system composed of a methylase and a nuclease. The methylase introduces specific modifications on the organism’s DNA, thereby marking it is as self. DNA lacking those modifications, i.e., DNA of foreign origin, will be cleaved by the nuclease. All four types of RM systems known to date are present in *S. aureus* [12]. Another, highly specialized phage defense mechanism is present in the form of staphylococcal pathogenicity islands (SaPIs) [13]. These mobile genetic elements interfere with the packaging of phage DNA in the late phase of infection, instead packaging and thereby disseminating copies of themselves. However, a small percentage of phage particles are still produced normally, leading to a reduced load of phage progeny instead of a total block. It has been implied that this may be an advantage to *S. aureus* as a species as it facilitates gene transfer [14]. Akin to abortive infection mechanisms, phage resistance by SaPI includes the lysis of the infected cell [13].

*S. aureus* is known to have a rather large accessory genome that can make up as much as 25% of total genome size [8]. We therefore hypothesize in this study that *S. aureus* may be carrying accessory genes that encode various mechanisms that are geared toward phage resistance. The presence of such mechanisms may hamper the efficacy of phage therapy, and it is therefore important to study these in order to perform optimization of phages used for treatment. With the advent of affordable high-throughput sequencing methods, it is now becoming possible to determine the whole genome sequences of the infecting strain in a clinical setting, making them accessible to this kind of investigation.

The relationship between *S. aureus* and its phages is intricate. A large proportion of *S. aureus* virulence factors are phage-encoded [8], and phages are the major agents of horizontal gene transfer in this species [11]. Furthermore, *S. aureus* is known to harbor prophages with a very high frequency, as detailed in a review by Lindsay in 2010 that states that all *S. aureus* sequenced up to that point contained at least one prophage [15]. In accordance with that, there is a sizeable body of research into staphylococcal phages, their genomes, their influence on their host’s evolution, and their contribution to *S. aureus*’ virulence (see for example [8,14,16]). Furthermore, phage susceptibility patterns have been used to classify *S. aureus* before the advent of molecular typing methods [17]. Despite that, there is a distinct lack of studies investigating the genetic basis for phage susceptibility and resistance in *S. aureus* from the host perspective, in particular with regard to whole genome approaches as opposed to studies focusing on single loci.

In this study, we seek to elucidate the interplay between *S. aureus* and therapeutic phage preparations. To do so, we have tested the susceptibility of a collection of clinical MRSA isolates towards a collection of staphylococcal phage preparations from HI. Both the bacterial and phage collections we used are of great relevance to the phage therapy efforts, since the phages are either already in use or under consideration for experimental therapy in accordance with European Union (EU) rules concerning compassionate use. Furthermore, the bacterial isolates were provided by Hvidovre Hospital in Hvidovre, Denmark and were obtained from patients showing complicated nosocomial MRSA infections. This strain set represents the most prevalent clonal complexes observed in Denmark. MRSA is predominantly imported, making the collection very diverse [18]. However, it is not representative of MRSA in all localities. The genomes of the bacterial strains were determined by whole genome sequencing and through employing a number of bioinformatics tools and machine-learning methods. We attempted to shed light on the genes of MRSA that play a role in determining the susceptibility or resistance towards phages. A similar approach but with different methodology was proposed by Allen et al., who tested for associations between phage and antibiotic resistance profiles with phylogenetic similarity in *E. coli* [19].

In this way, we aim to contribute to the development of predictive tools of phage susceptibility in the phage therapy–targeted bacteria and ultimately to devising strategies for the prevention, delay, or circumvention of phage resistance in a phage therapy setting.

## 2. Results

### 2.1. General Results of the Susceptibility Testing

A total of 207 MRSA strains were successfully tested for susceptibility to 12 phage preparations. The ratio of susceptible to resistant strains differed between the preparations. Note that phage preparations were standardized to routine test dilution (RTD). The percentage of susceptible strains ranged from 19% to 68%, as can be seen in Table 1. We have chosen to regard both weakly susceptible and resistant reactions as negatives for the modelling. We did not observe a large difference in efficacy between single phage preparations and mixtures. 

### 2.2. Genetic Diversity of the Strain Collection

**Figure 1:**
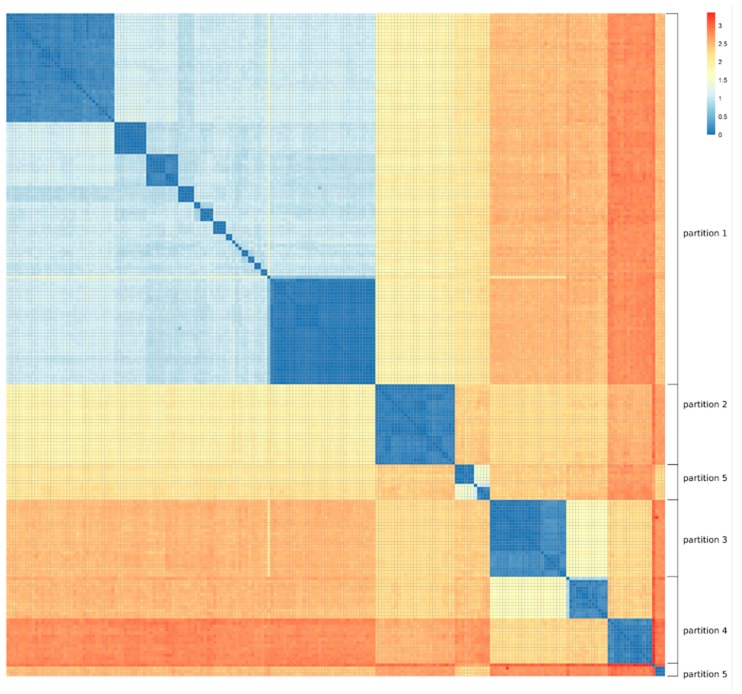
All-against-all matrix of the genetic distance between the 207 methicillin-resistant *Staphylococcus aureus* (MRSA) strains used for this study. Distance is calculated as 1-orthoANI and represented as color, where blue corresponds to lower and red corresponds to greater distance. The assignment of strains to partitions is marked on the right margin.

Genetic distance between the MRSA strains was measured as 1-orthoANI (see Methods), and the result is depicted in form of a heatmap in Figure 1. This figure reveals a clear clustering of strains into groups with high identity, which follows the established clonal complexes and sequence types of *S. aureus* [20]. Based on this clustering, the strains were split into five partitions by visual inspection.

Partition 1 is substantially larger than the other four. This is due to the fact that the strains belonging to clonal complexes CC1, CC5, CC8, and CC80 have a high degree of identity to each other, compare large blue area in the upper left corner. Partitions 2 and 3 are well defined, encompassing CC22 and CC30, respectively. Partition 4 is made up of CC45 and CC398. CC398 is known for its prevalence in swine and cattle. Those strains are genetically distant from the rest of the strains, though there is some degree of similarity to CC30. Partition 5 is composed of two clusters of related strains, as indicated in Figure 1. It contains a number of rarer CCs that also show a comparatively high distance in terms of orthoANI to the rest of the data set. 

### 2.3. Identification of Gene Families

**Figure 2:**
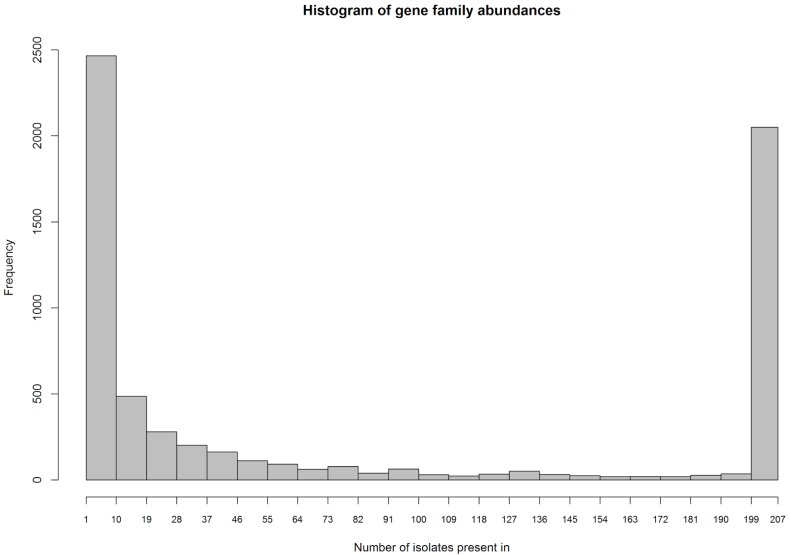
Abundance of gene families in the 207 strains. The peak depicted in the histogram is slightly higher than the number of housekeeping genes, 1.777, since the bin is wider than 1.

When predicting and clustering genes, we identified a total of 6419 gene families in the MRSA strain dataset. The distribution of these gene families across the 207 MRSA strains can be seen in Figure 2, which shows a histogram of abundances of the gene families. Here, 1777 gene families were identified in all 207 strains. These are the housekeeping genes. Furthermore, there is a heavy tail of gene families that were only observed in few strains (left side of the histogram).

#### 2.3.1. *p*-Value Distribution from Association Tests

Models are set up in five-fold cross validation frameworks (see Methods Section 4.5). For each cross validation fold, each gene family was assigned a *p*-value calculated from its corresponding contingency table estimated once from the original data and once from permuted data. We chose here to illustrate results for phage P4/6409 as it was representative of the other phage preparations.

**Figure 3:**
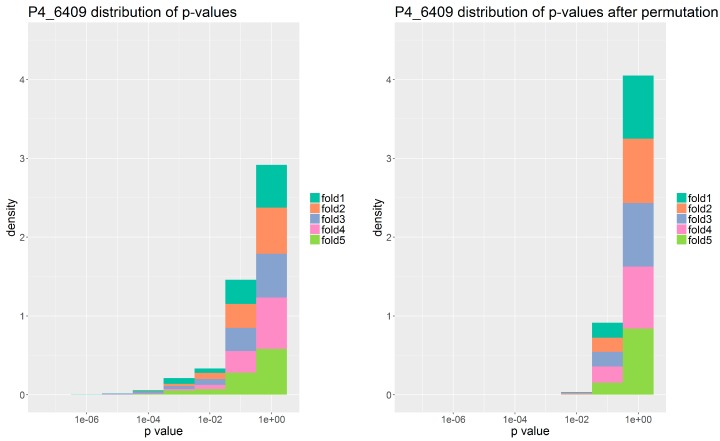
Stacked histogram of *p*-value distributions across the five folds for the interaction with phage P4/6409. The density is shown instead of counts to account for fold 1 having a 100 times less *p*-values compared to the other folds, since it does not include partition 1 and therefore did not need to be subsampled. **Left**: Real data. **Right**: Permuted data.

When plotting the distributions of these *p*-values, see Figure 3, we can make several observations. 

(a) In most phage interactions, there is a small tail of gene families with very low *p*-values, while the majority of gene families have non-significant *p*-values.

(b) In the permuted data, this tail vanishes, as was to be expected. We also observed that the *p*-value distributions of phages 1N/80, A3/R and cocktail MS-1 resemble those of the permuted data much more than those of the real data (see Supplementary Figure S1). This indicates there were not enough positive examples of lysed strains to produce a signal that is distinguishable from random.

Based on these observations, a *p*-value threshold of 0.01 or lower was implemented to admit gene families to the first step model. As seen in Table 2, the number of gene families picked by enrichment varied both by fold as well as by phage. In preparations 1N/80, A3/R, and mix MS-1, the number of gene families picked was very low. Further, as expected, we find that no or only very few gene families are selected when analyzing the permuted data.

#### 2.3.2. Refinement Based on Regression Models

In the second step of feature selection, we employed linear regression models fitted using Ridge regression. An internal cross validation was used to identify the optimal parameter for the Ridge penalty lambda. The optimal lambda penalty value across the different folds in the cross validation were comparable, indicating that the models are robust, though the size of the feature space varies (see Supplementary Figure S2).

We next required that a gene family should have absolute regression weights greater than 0.01 in at least three of the five partitions to have passed a second selection step. The number of gene families selected in this manner is listed per phage on the right side of Table 2. We term this the set of significant gene families for a certain phage. The number of significant gene families in interaction with phages 1N/80, A3/R, and mix MS-1 was too small to train a final model. For the remaining phages, the amount of significant gene families varied between the different phages, though the sets were comparable in size, with the smallest comprising 13 and the largest 80 gene families (see Table 2). In total, there were 167 significant gene families. When performing the same procedure on permuted data, significant gene families could only be identified in four phages, and a final model could only be trained for two.

#### 2.3.3. Final Model

**Figure 4:**
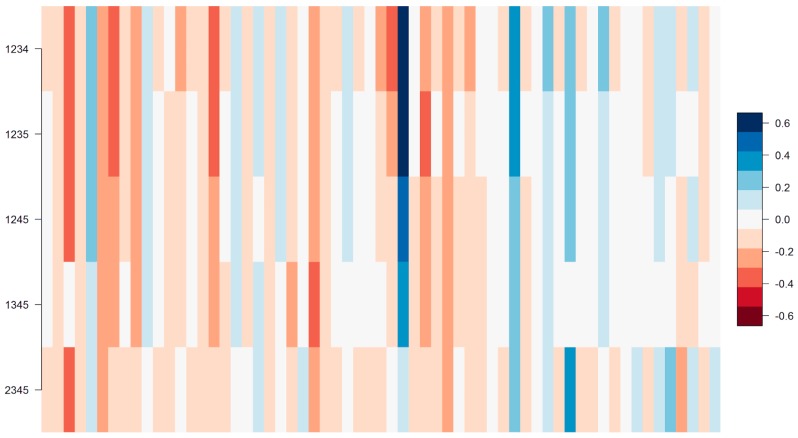
Heat map of the regression weights for the final model of phage P4/6409. Columns are gene families, rows are cross validation folds. The color indicates the value and direction of each weight, with blue being strongly positive and red being strongly negative. Weights with low values are white. Results were comparable for other phages with the exception of 1N/80, A3/R, and mix MS-1 (see Table 2).

Final models were next retrained including only the significant gene families passing both steps of feature selection (low association *p*-values and high regression weights) as input features. Plots of the regression weights assigned by those final models showed the direction of weights to be consistent across folds, i.e., gene families are consistently found to have either positive or negative weights across all of the five partitions. This is depicted for the example of phage P4/6409 in Figure 4. Results for other phage preparations were comparable.

Out of all the 167 gene families, a total of 97 increased phage resistance, 62 increased phage susceptibility, and eight were ambiguous, meaning that they increased resistance to some phages but susceptibility to others. This further shows that the vast majority of significant gene families identified were consistent in their direction of influence across all 12 tested phage preparations.

### 2.4. Functional Annotation of the Significant Genes

**Figure 5:**
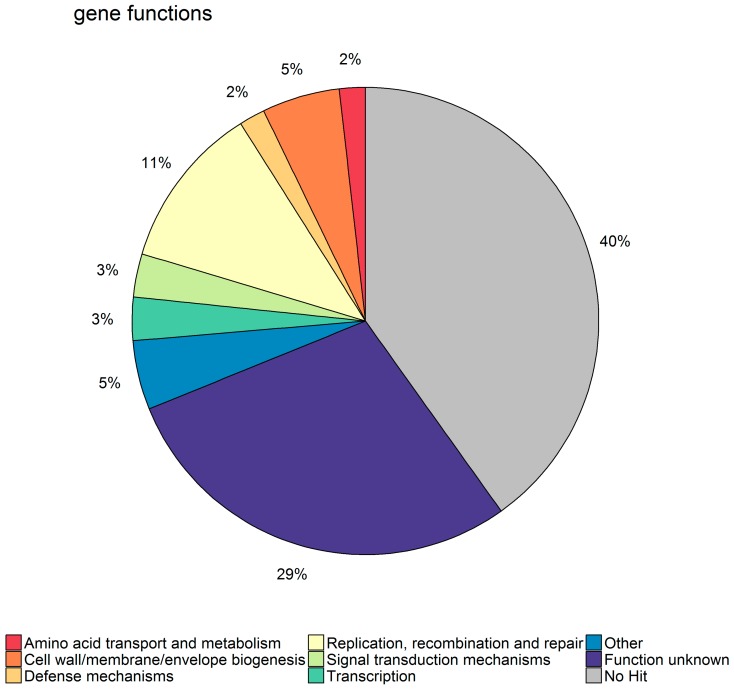
Functional annotation categories of the eggNOGs matching to the set of significant genes across all nine phages.

We further sought to characterize the function of the identified significant gene families by comparing them to the eggNOG database. The distribution of functional annotation terms identified for the full set of significant genes is shown in Figure 5 and shows that it was possible to identify a match in eggNOG for only 60% of gene families. Most genes had either no hit in the eggNOG database or a hit to a NOG of unknown function. 

Case-by-case inspection of the functional annotation terms retrieved from both RAST and eggNOG for the 167 significant gene families identified 13 gene families that have terms directly related to phages, while another 18 were related either to other mobile genetic elements such as genomic islands and transposons or to processes associated to them such as transposase activity. Of these, three gene families have homologs found in SaPIs, which are a phage defense system of *S. aureus* [13]. Four additional gene families appeared to be part of restriction-modification systems and six had hits to transcriptional regulators.

Out of these groups, the gene families related to restriction-modification systems and SaPIs were found to consistently be associated with resistance to phage infection (as measured by the sign of the weights in the final model described earlier), as can be seen in Supplementary Table S1. Of the gene families associated with transcriptional regulators, five were found to increase phage resistance, while one was found to increase susceptibility. The gene families related to phages and mobile elements encompass both gene families promoting resistance and families promoting susceptibility, further pointing to the complexity of the host–phage interaction. The full list of annotation terms for all significant gene families can be found in the Supplementary Table S1, together with the gene family’s average regression weight across the five cross validation folds per phage.

We have estimated cumulative density functions (CDF) for each eggNOG category from the full gene set and next evaluated which functional categories in the significant gene set were enriched or depleted. With a threshold of *p* = 0.05, we found that categories “No hit” and “Replication, recombination, and repair” were enriched, while “Post-translational modification, protein turnover, and chaperones” and “Inorganic ion transport and metabolism” were depleted (see Supplementary Table S2). 

### 2.5. Overlap of Significant Gene Family Sets

**Figure 6:**
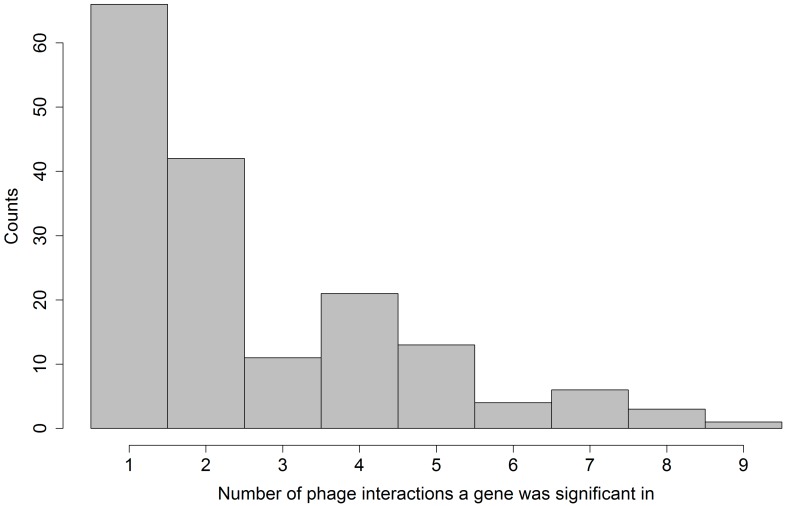
Histogram depicting the number of phage models where a given gene family was identified significant.

We further analyzed the overlap between the significant gene family sets found for each phage model. Figure 6 shows a histogram of the number of phage models where a given gene family was identified as significant. It clearly presents that very few significant gene families are shared by many phage models, and only one is shared by all nine. The majority of significant gene families have been observed in interaction with only one or two different phages. This in turn means that each of the phages we tested has a distinct and specific interaction with our bacterial strain set, since different genes in the bacterial host dictate whether infection will be successful.

Further, the significant gene families of the three cocktails are not a linear combination of the sets identified for their component phages, though there is a sizeable overlap (data not shown).

There were four gene families found significant in at least eight phage models. They are listed in Table 3, along with their direction of influence and the annotation and category of their matching eggNOG, if any. Out of the four, three increase resistance to phage, while one was ambiguous in its direction of influence. Two gene families had no hit in the eggNOG database and one was categorized as being of “unknown function”. We were therefore unable to deduce a possible function for them though they appear to be of great importance for phage susceptibility. One, cluster 3112, appears to be involved in regulation of transcription and signal transduction that may play a role in host takeover. There were no direct indications for how exactly those gene families effect their influence biologically, but it is evident from the models that they do.

## 3. Discussion

In this study, we sought to model the host-genetic determinants of MRSA phage susceptibility with a two-step logistic regression model fitted via ridge regression. We succeeded in building models of acceptable performance for nine of the 12 tested phage preparations with AUCs ranging from 0.65 to 0.87. By doing so, we identified 167 host gene families that influence *S. aureus*’ interaction with those nine phages. 

Our dataset is, with 207 observations, rather small for this type of analysis, since there are many more covariates—i.e., gene families—than observations. We have addressed this by building a two-step model and including a filtering step based on *p*-values, thereby greatly reducing the number of covariates going into the analysis. As biological entities are shaped by evolution, the strains share some degree of relatedness, and the testing results are not completely independent observations. We have partitioned the data according to phylogeny in a way that ensures highly similar strains are located to the same partition. Doing that ensures that the observations we are aiming to predict are more independent from the ones we feed into the model during training. The partitioning was maintained at all steps, ensuring that data from highly similar strains was never used to predict the outcome. Furthermore, there was an uneven partitioning of the data due to a high percentage of strains from two very related sequence types, which may lead to bias. The challenge of uneven partitions was addressed by subsampling the oversized partition 1 so we could obtain a realistic distribution of *p*-values for the association of all genes to the observed phenotype. Finally, our set of strains with its composition of clonal complexes is specific to Denmark [18]. It is not necessarily representative of *S. aureus* populations observed in different settings.

It should further be noted that our approach can only identify gene families that are part of the accessory genome, since the first selection step is based on differential abundance of those gene families in susceptible vs. resistant strains. Furthermore, this analysis does not consider point mutations as far wild type and mutant version of a gene are more than 90% identical, since we have clustered genes into families with that threshold. 

Regarding the electronic gene family annotation, we were able to identify four gene families related to restriction-modification systems and three related the genes found in SaPIs, all of which increased the resistance to phage as expected. Further, six of the significant gene families were related to transcriptional regulation, which fits well with the fact that phages try to shut down host transcription during takeover.

A multitude of gene families found appear to be mobile elements of some kind. Interestingly, Ram et al. have stated that “Most genes involved in phage resistance are carried by plasmids and other mobile genetic elements, including bacteriophages and their relatives” [14], though this statement is quite possibly related to SaPIs and phage-inducible chromosomal islands (PICIs) in general. Those mobile element related gene families had varying direction of influence. They may be related to the interplay of integrated prophages and external phages, which can either complement each other or oppose each other. An integrated prophage may for example protect from further infection via a principle known as superinfection-exclusion [21]. For a large proportion of the significant gene families, however, no hit could be found in the eggNOG database, and of those that had a hit, the most common category was “Function unknown”. This may be due to the fact *S. aureus* has a large accessory genome that is made up mostly of different types of mobile genetic elements, among them prophages, that are highly diverse and not well characterized [8]. We have not determined whether either the gene families with hits to phage related proteins or those without hits or with hits to proteins of unknown functions are parts of integrated prophages. Identification of the prophages present in our strain set could add to the interpretation of the analysis; however, it is out of the scope of this study.

We also found that there is only a minor overlap between the sets of significant gene families identified for different phages. This means that each phage had a different and specific interaction with the set of bacterial strains. 

Further, we found that generally more gene families promoted resistance than susceptibility. Among the four gene families that were found significant in interaction with at least eight different phages, three promote resistance, and one was ambiguous (see Table 3). This overrepresentation of gene families promoting resistance was expected, since in our set-up resistance to phage can more easily be explained by a gain of function model, meaning the gaining of a defense mechanism of which there are plenty found in nature. We were unfortunately unable to identify the nature of the defense mechanism in most resistance promoting gene families from electronic annotation alone. 

Conversely, a gain in susceptibility linked to the presence of a certain gene family is more difficult to explain. The most ready interpretation is that these gene families somehow improve conditions for the phage. The observation can also be explained by integrated prophages that may become activated upon infection or stress caused by the adsorption of an external phage and then lyse their host after completing the lytic cycle. Since the products of the bacterial lysis by the phages were not sequenced, we cannot say whether the external, therapeutic phage or an integrated prophage is the agent of the lysis. Intriguingly, evidence of an interplay between virulence and phage resistance has also been shown. Laanto et al. report that after co-cultivation with lytic phage, strains of the fish pathogen *Flavobacterium columnare* that have acquired phage-resistance have also lost their virulence compared to phage-sensitive paternal strains [22]. Similar observations have been made for *S. aureus* by Capparelli et al. [23], who show that phage-resistance is associated with reduced fitness. Accordingly, the opposite correlation may hold as well, meaning that genes associated with higher virulence and host fitness may at the same time effect higher susceptibility to phages. As our strain set was isolated from patients displaying severe *S. aureus* infections, it is conceivable that these strains are both very virulent and of high fitness. 

In conclusion, we have shown that while our methodology does not have predictive power, it allows for the association of the observed phenotype with the genetic background, thereby producing interpretable results that can be used for gene function discovery. This type of analysis, which combines phenotypic and whole genome sequencing (WGS) data, can be used to identify genetic determinants of observed bacterial phenotypes in other settings as well and is expected to be a useful tool in future analyses of phage-host relationships

## 4. Materials and Methods

### 4.1. Collection of Clinical MRSA Strains Used for Susceptibility Testing

The collection of 207 MRSA strains tested in this project as well as their whole genome sequences (WGS) were obtained from the Clinical Microbiology Department of Hvidovre Hospital, Hvidovre, Denmark. The strains originate from patient samples. They were selected to represent a broad genetic diversity of the more than 5000 WGS MRSA from Hvidovre Hospital. The fasta sequences of the 207 selected strains have been submitted to the European Nucleotide Archive (Hinxton, Cambridgeshire, UK) [24] with the accession numbers ERZ485118–ERZ485325. They can be viewed under the link: http://www.ebi.ac.uk/ena/data/view/<Accession Numbers>.

Although no methicillin-sensitive (MSSA) strains were included in the study, we nonetheless chose MRSA strains of the spa-types that are common in MSSA infections [25]. Spa-typing is a single-locus classification scheme for *S. aureus* based on the polymorphic region in protein A [26]. We included MRSA strains positive for PVL and containing *mecC*. All inclusion criteria are listed Supplementary Section 1 ‘List of inclusion criteria for MRSA strains’ and the properties of selected isolates can be found in the Supplementary Table S3.

### 4.2. Collection of Phages Used for Susceptibility Testing

A total of 12 therapeutic staphylococcal phage preparations were used for susceptibility testing. They contain phages which are part of the proprietary collection of therapeutic phages used by the phage therapy unit of the Hirszfeld Institute of Immunology and Experimental Therapy of the Polish Academy of Science in Wroclaw (HI) [27]. Nine of the preparations are monovalent phage lysates: 1N/80, 676/F, 676/T, 676/Z, A3/R, A5/L, A5/80, P4/6409, and phi200/6409. Crude phage lysates were prepared according to the modified method of Ślopek et al. [9]. Six of those phages (1N/80, 676/Z, A3/R, A5/80, P4/6409, and phi200/6409) were sequenced and confirmed to be obligatory lytic and belonging to a Twortlikevirus genus of a Spounavirinae subfamily of Myoviruses. A detailed report on characteristics of these six phages can be found in Łobocka et al. [28]. All monovalent phage preparations were standardized to routine test dilution (RTD) and had a titer between 10^6^ and 10^8^. RTD is the highest dilution that still gives confluent lysis on the designated propagating strain of *S. aureus* [17] and the standardization method of choice at HI. 

MS.1, OP_MS.1, and OP_MS.1_TOP were equal mixtures of A5/80, P4/6409, and 676/Z phages prepared at the Institute of Biotechnology, Sera and Vaccines BIOMED S.A. in Cracow, Poland. MS-1 phage cocktail lysate contained each component phage in a titer no less than 5 × 10^5^ pfu/mL, OP_MS-1_TOP cocktail of purified phages was suspended in phosphate buffered saline containing each phage at no less than 10^9^ pfu/mL [29], and OP_MS-1 phage cocktail had similar characteristics as OP_MS-1_TOP but contained up to 10% of saccharose as a phage stabilizer.

### 4.3. Susceptibility Testing Procedure

Testing for phage susceptibility was performed as described by Ślopek et al. [30]. In short, 50 μL of phage preparation was applied onto a fresh bacterial lawn from day culture and the results were assessed the next day following 6 h incubation at 37 °C. 

Results were assessed according to a 7-point scale as described by Ślopek et al. [30] and shortly summarized in the  supplement Section 9 ‘Details on susceptibility testing as described by Ślopek et al.’ Results were further discretized into two levels: “susceptible” and “resistant”. The “susceptible” label was applied to the two strongest reactions, resulting in confluent or semi confluent lysis. According to standards applied at the Bacteriophage Laboratory of the HI, those two levels enable the phage procurement for therapeutic phage preparation. All other weak reactions as well as a negative reaction and opaque lysis were regarded as “resistant”. Susceptibility testing results in these two levels, as used for the modelling, can be found in Table 1, while Supplementary Table S4 details results in three levels: resistant, weakly susceptible and strongly susceptible.

The full set of 207 strains was challenged with each of the 12 phage preparations. We call the result of susceptibility testing to a preparation the “interaction” of our strain set with said phage. 

### 4.4. Data Partitioning

For the purpose of modelling the phage response from the genomic composition of the bacterial strains, the 207 MRSA strains were divided into five partitions. This division was based on the orthogonal average nucleotide identity (orthoANI) as described by Lee et al. [31]. OrthoANI is suitable for creating a distance matrix, because it is a symmetric measure of distance, unlike the traditional ANI. Calculations were performed on all pairs of strains with the standalone tool OAT by Lee et al. Distances were subsequently calculated as 1-orthoANI, and a heat map was generated that can be found in Figure 1.

The resulting heat map showed very clear clusters of closely related sequences. Partitioning was therefore done by visual inspection.

The partitions thus obtained were then used in a five-fold cross validation framework, i.e., four of them were combined into the training set, and one was left out for testing. This process was repeated five times so that each partition was in turn the testing set.

### 4.5. Model Framework

We sought to model a binary outcome (resistant/susceptible) based on weighted binary features (absence/presence of gene families). Logistic regression models were chosen for this task and set-up inside a five-fold cross validation. Each cross validation fold was trained using a Ridge regression to avoid overfitting. A nested cross validation was used to identify the optimal parameter for the Ridge penalty lambda. 

Due to challenges posed by the large feature space, the modelling was further split into a two-step process: a first-step model in which we performed feature selection by association testing, and a second-step model whose features were selected based on the regression weights obtained from the first model. The following sections describe details of each modelling step.

### 4.6. Feature Selection by Association Testing

The genetic background of the MRSA strains was established by first predicting genes and performing functional annotation through the RAST service [32] for all 207 strains. The predicted genes were then clustered with cd-hit [33] using a cutoff of 90% on global sequence identity, word size 5 and the -g 1 option to cluster with the best match instead of the first match. This resulted in a total of 6.419 gene families in the 207 MRSA strains.

Next, the feature space, i.e., the number of gene families included in the model, was reduced by removing gene families with limited power for distinguishing susceptible from non-susceptible bacterial strains. This was done by constructing 2 × 2 contingency tables as illustrated in Supplementary Table S5, and from these tables calculating a *p*-value to each gene family in each phage interaction using Fischer-Boschloo’s exact unconditional test. We then imposed a threshold of 0.01 on the *p*-value for the gene family to be admitted to the second step of modelling.

As can be seen in Figure 1, one of the partitions was significantly larger than the other four. This obliged us to employ bootstrapping in every fold that included partition 1 so as to not bias the feature selection on partition size. Details to this can be found in the Supplementary Section 10 ‘Details on Feature selection by association testing’.

### 4.7. Feature Selection by Regression Weights

Due to the five-fold cross validation setup, each gene family was assigned five regression weights for interaction with each phage preparation. These may be NA (not applicable) if the gene family was not chosen by association testing for that fold. Weights can be either positive or negative. As we chose to model susceptibility as the positive outcome and resistance as the negative outcome, this means that positive weights point towards increased susceptibility, while negative weights point towards increased resistance. 

We hypothesized that gene families with a high weight across many folds drive the response to this particular phage. Therefore, we next trained and tested a second five-fold cross validated regression model with only the genes that (1) were significant according to the Fischer-Boschloo’s test (*p* ≤ 0.01) and (2) had absolute regression weights greater or equal to 0.01 in at least three folds in the first regression model. We term the gene families selected in this fashion the set of significant gene families. They are the main focus of this study as they are thought to be driving the response to the tested phage preparations.

In order to verify that the set of gene families we identified were indeed descriptive of the phage susceptibility and not an artifact of overfitting, we repeated the model construction and feature selection with shuffled target values. That is, we randomly associated susceptibility outcomes and bacterial genomes while keeping the ratio between susceptible and resistant as in the original data. We then re-ran the modelling and evaluated the predictive performance and the number of predictive gene-families identified.

### 4.8. Assignment of eggNOGs

We compared each selected gene family to the eggNOG database [34] by using the eggNog-mapper available on their webpage. eggNOG is a database of non-supervised orthologous groups (NOG) of proteins based on the clustering of the 9.6 million proteins from 2031 genomes. Each NOG has only one annotation term compiled from the integrated and summarized functional annotation of its group members, as well as being part of a broader functional category. EggNOG was chosen primarily because of this functional category assignment that allows a broad overview of the functions present in a set of genes.

To estimate whether the observed distribution of functional categories in the set of significant gene families was different from what could be expected by chance, we employed the cumulative density function (CDF). We first drew 10,000 random subsamples of the same size as the full set of significant genes families from the total set of 6419 gene families. From these data, we established an estimated cumulative density function (eCDF) for each functional category. We could then calculate likelihoods for each category of obtaining the actual observed frequency or lower or, conversely, the actual observed frequency or higher. 

## Figures and Tables

**Table 1:** Wet lab results of susceptibility testing. All phage preparations were tested at RTD, see Methods. MS-1, OP_MS-1 and OP_MS-1_TOP are mixtures of P4/6409, A5/80 and 676/Z.

Phage Preparation	Percent Sensitive	Percent Resistant
1N/80	31.9%	68.1%
676/F	50.7%	49.3%
676/T	68.1%	31.9%
676/Z	40.6%	59.4%
A3/R	18.8%	81.2%
A5/L	47.3%	52.7%
A5/80	55.1%	44.9%
P4/6409	37.7%	62.3%
phi200/6409	44.0%	56.0%
MS-1	33.8%	66.2%
OP_MS-1	38.6%	61.4%
OP_MS-1 TOP	39.6%	60.4%

**Table 2:** Summary of the modelling results for real and permuted data. The “First Model” section reports the results of the first filtering procedure based of association analyses. The “Final Model” section gives the result of the second filtering procedure based on regression model fitting combined with consistency constraints. The area under the curve (AUC) is used as performance measure of the final model. The number of gene families selected given in the left part of the table is calculated as the average ± standard deviation across the five folds. If less than two gene families were selected based on regression weights, a final model could not be trained and the associated AUC is reported as NA (not applicable).

	First Model		Final Model			
	Real Data	Permuted Data	Real Data		Permuted Data	
**Phage Preparation**	No. of Gene Families Selected by Enrichment	No. of Gene Families Selected by Enrichment	No. of Gene Families Selected on Regression Weights	**AUC**	No. of Gene Families Selected on Regression Weights	**AUC**
1N/80	10 ± 16	0	2	NA	0	NA
676/F	222 ± 144	0	45	0.78	0	NA
676/T	361 ± 243	12 ± 11	79	0.87	3	0.63
676/Z	112 ± 87	11 ± 14	31	0.72	4	0.61
A3/R	13 ± 26	0	1	NA	0	NA
A5/L	184 ± 124	0	37	0.8	0	NA
A5/80	265 ± 148	0	80	0.78	0	NA
P4/6409	200 ± 137	2 ± 4	61	0.79	0	NA
phi200/6409	160 ± 138	0	56	0.79	0	NA
MS-1	6 ± 10	0	0	NA	0	NA
OP_MS-1	86 ± 78	0	29	0.65	0	NA
OP_MS-1_TOP	54 ± 52	1 ± 1	13	0.67	0	NA

**Table 3:** Predicted functions of the gene families found significant in interaction with eight or more phages.

Gene Family ID	Times Observed	Increases	eggNOG Annotation	eggNOG Category
cluster_1791	9	Resistance	-	No Hit
cluster_389	8	Resistance	-	Function unknown
cluster_3112	8	Resistance	Transcriptional regulator	Transcription
cluster_3992	8	Ambiguous *	-	No Hit

* This gene family always confers phage resistance except in one interaction in which it confers susceptibility.

## Supplementary Materials

The following are available online at http://www.mdpi.com/2079-6382/7/1/9/s1: Figure S1: *p*-Value distributions of gene enrichment analysis on phage preparations 1N_80, A3_R and cocktail MS-1. Figure S2: Plot of the cumulative mean square error of the inner cross validation vs. strength of the ridge penalty. Table S1: List of all significant gene families along with their functional annotation terms. Table S2: Probabilities of observing a given prevalence per functional category based on the cumulative density function. Table S3: List of MRSA strains included in the test set and their properties. Table S4: Detailed phage typing results. Table S5: Layout of the contingency tables. The supplement further contains sections one the following: Details of inclusion criteria for MRSA strains. Details on susceptibility testing as described by Ślopek et al. Details on Feature selection by association testing.
