# Supplementary material for: Use of a Regression Model to Study Host-Genomic Determinants of Phage Susceptibility in MRSA
Source: Antibiotics (Basel). 2018 Jan 29;7(1):9. doi: 10.3390/antibiotics7010009 (PMC5872120; doi:10.3390/antibiotics7010009)
Supplement: Supplementary file 1 [file antibiotics-07-00009-s001.zip › Suplementary-final/figure_s1.docx]

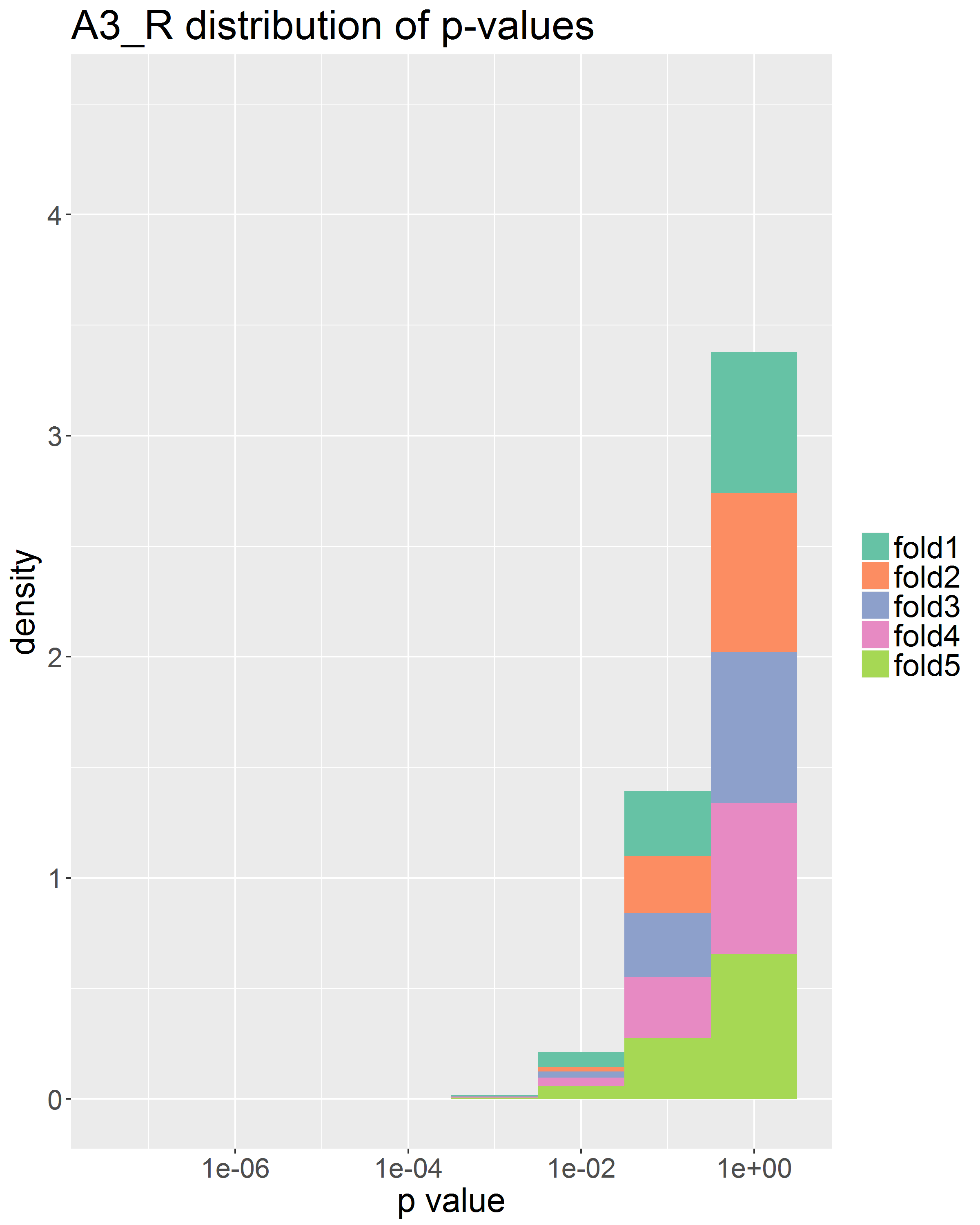

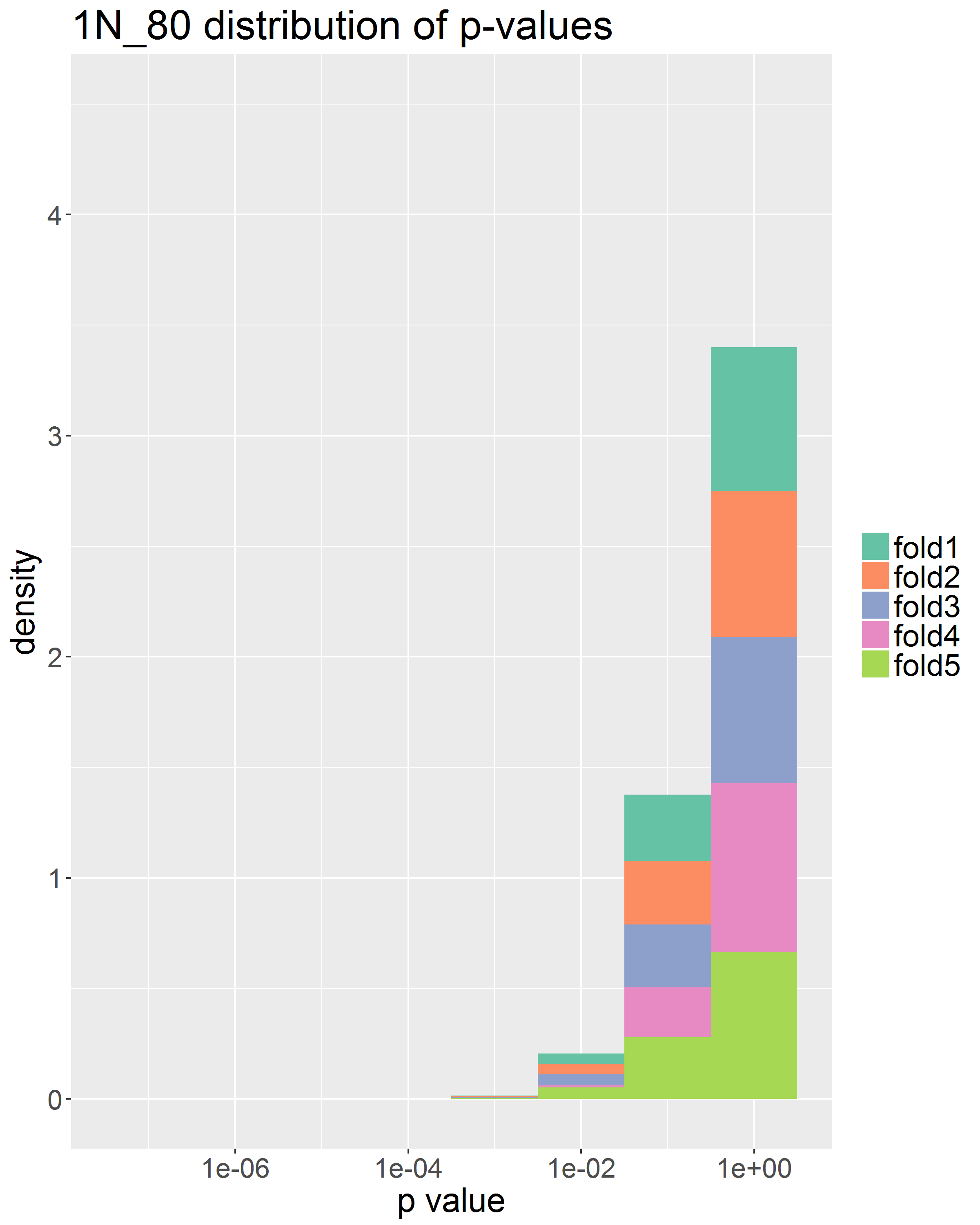
a) b)


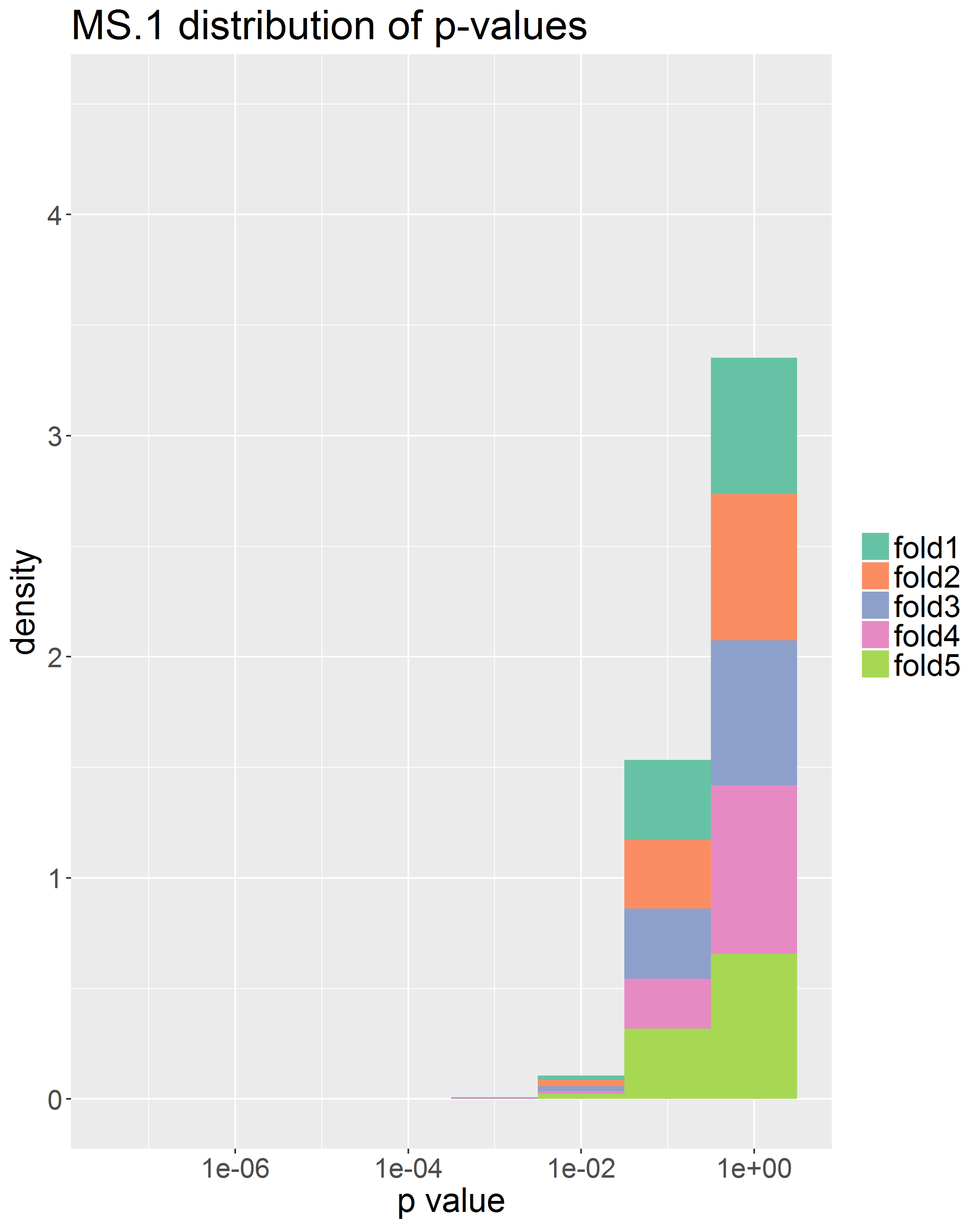
c)

Supplementary Figure S1. P-value distributions of phage preparations a) 1N_80 and b) A3_R and c) cocktail MS-1. It can be seen that there is no tail of low p-values as observed for the other phages (compare Figure 3) and the distributions resemble more closely that of the permuted data for the other phages.
