# Supplementary material for: Use of a Regression Model to Study Host-Genomic Determinants of Phage Susceptibility in MRSA
Source: Antibiotics (Basel). 2018 Jan 29;7(1):9. doi: 10.3390/antibiotics7010009 (PMC5872120; doi:10.3390/antibiotics7010009)
Supplement: Supplementary file 1 [file antibiotics-07-00009-s001.zip › Suplementary-final/Supplement.docx]

The following supplementary files are available:

1) List of inclusion criteria for MRSA strains.

All strains originate from patient samples. Strains were included in the study if they met one or more of the following criteria:

1. Having one of the ten most common spa types that occur in Methicillin-sensitive Staphylococcus aureus infections.
2. Positive for Panton-Valentine leukocidin (PVL), a major virulence factor of S. aureus that is common in community acquired strains[1].
3. Positive for mecC, a divergent methicillin resistance gene discovered in 2011. Strains with this gene may be miss-classified as methicillin-sensitive due to the absence of mecA, the stereotypic resistance factor [2].
4. Being of a rare clonal complex. This criterion was included to obtain a more diversified strain collection.
5. Being of one of the major clonal complexes prevalent in Europe, which arecc22, cc30, cc45 [3]
6. Being of clonal complex 398, which is typically livestock associated
7. Additionally, strains where the sequencing data was of good quality were preferred over strains with poor quality sequencing data.

2) Supplementary Figure S1. P-value distributions of gene enrichment analysis on phage preparations a) 1N_80, b) A3_R and c) cocktail MS-1. It can be seen that there is no tail of low p-values as observed for the other phages (compare Figure 3) and the distributions resemble more closely that of the permuted data for the other phages.


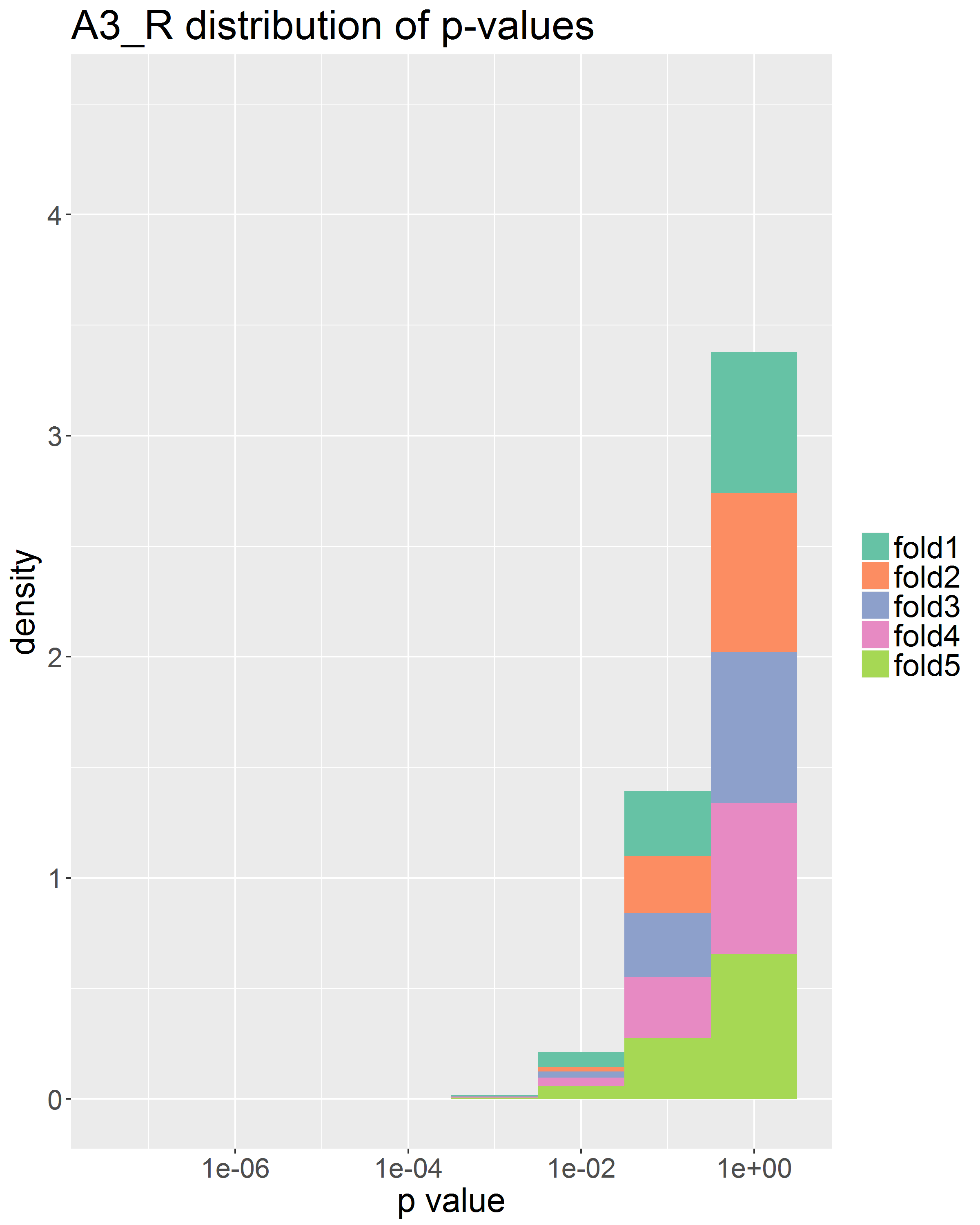

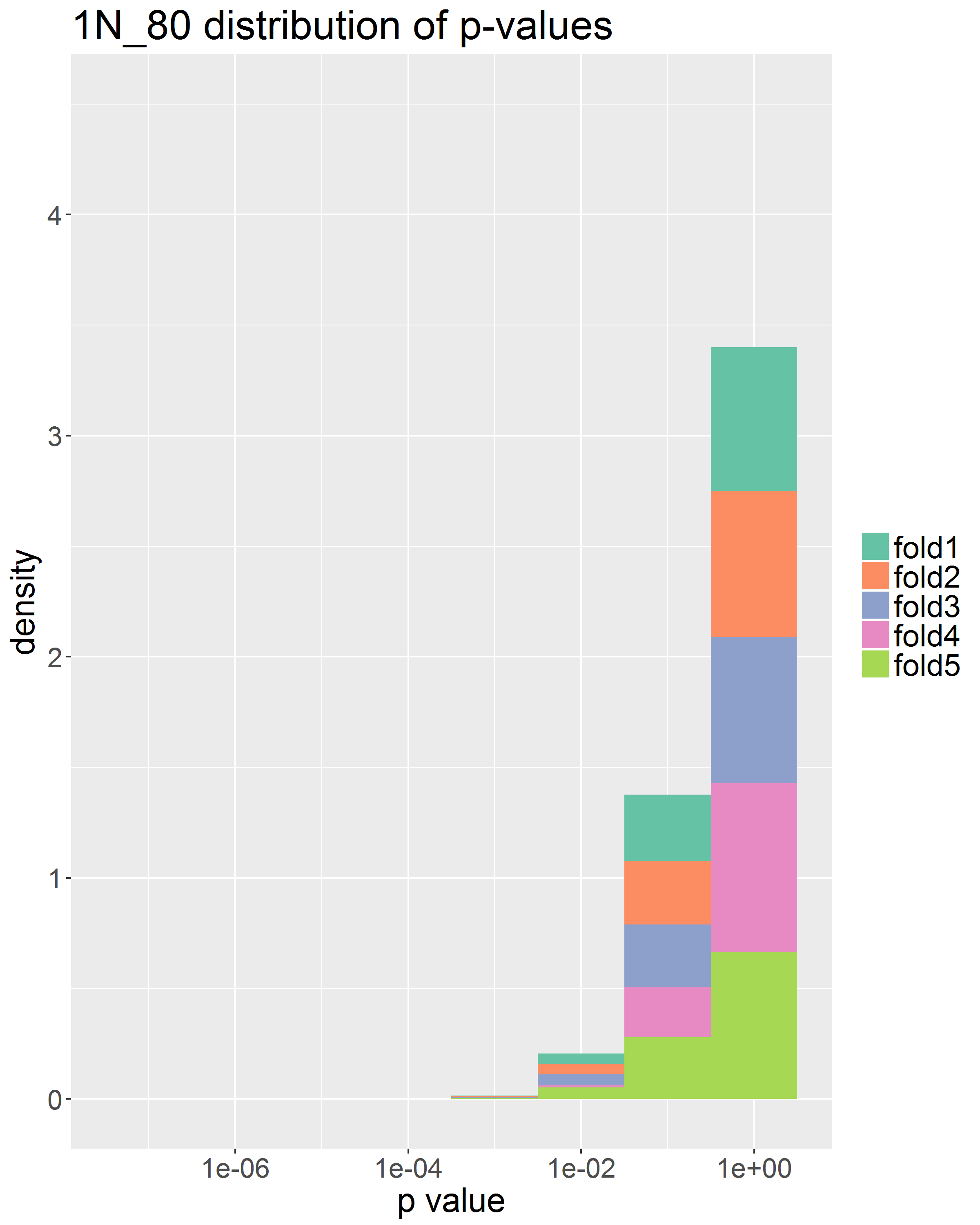
a) b)


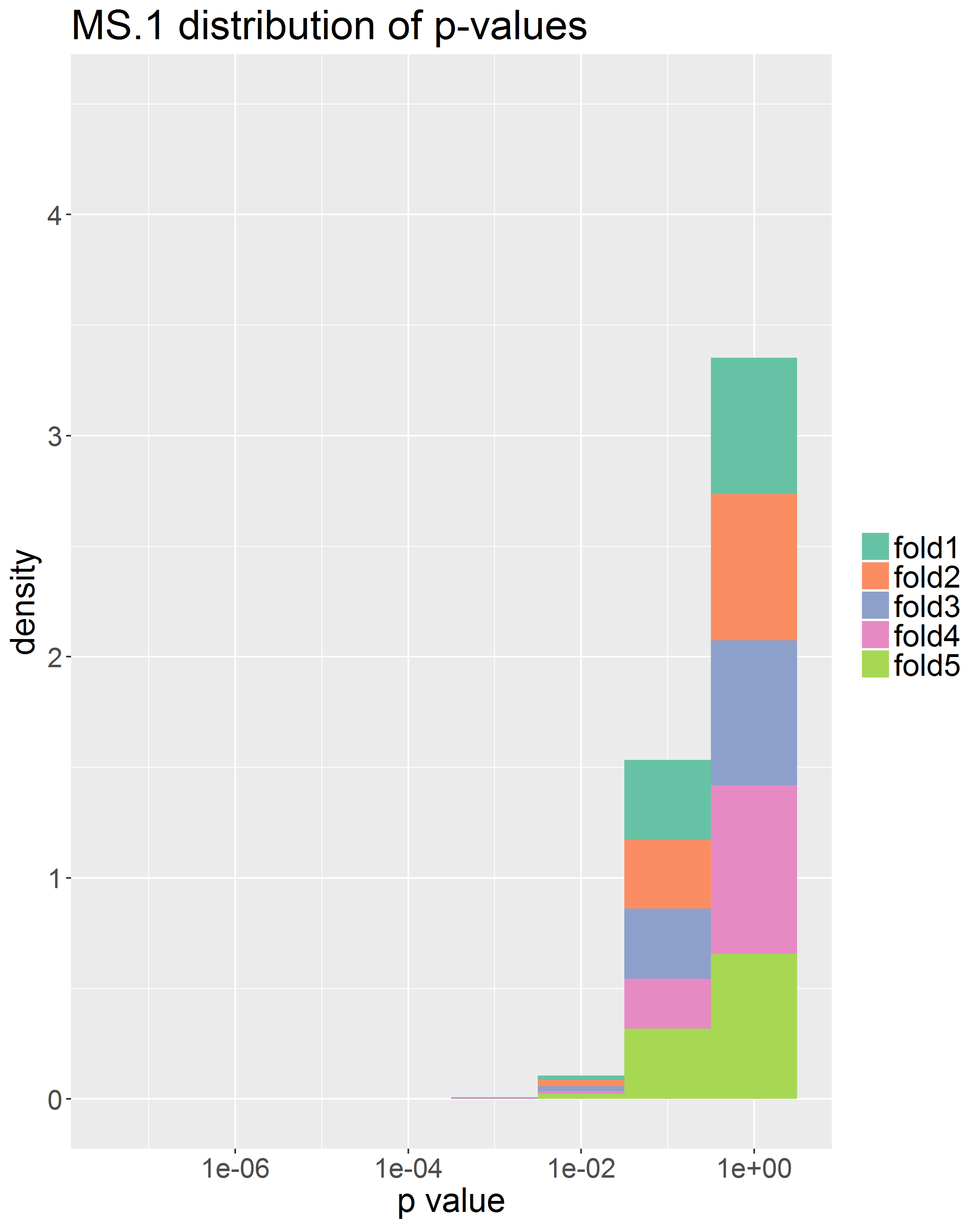
c)

3) Supplementary Figure S2. Cumulative mean square error of the inner cross validation vs strength of ridge penalty per outer fold for the model of phage phi200/6409. It can be seen that the minimum error coincides at similar lambda values for the five folds. Other phage models behaved in comparable fashion.


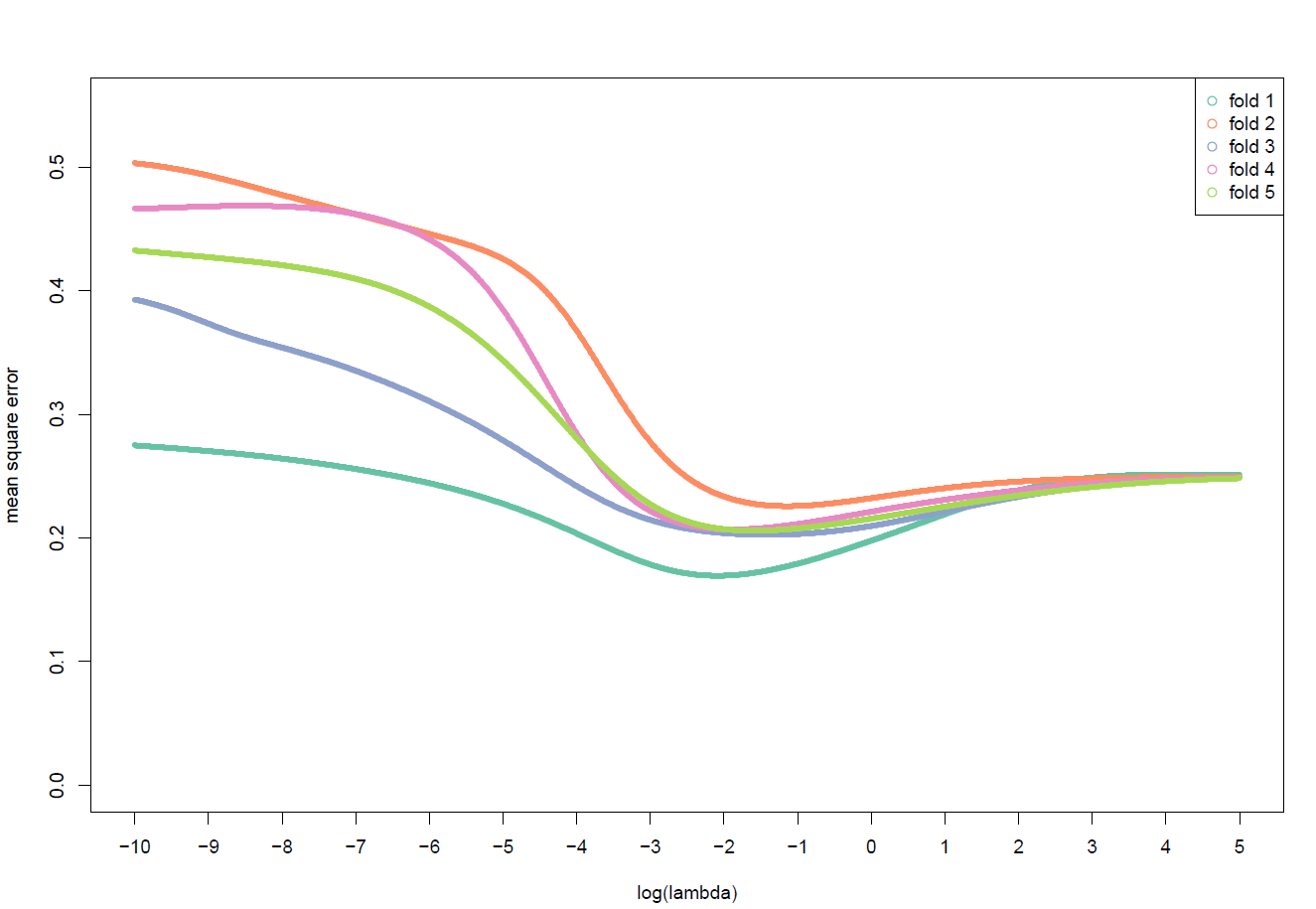


4) Supplementary Table S1. List of all significant gene families along with the functional annotation terms retrieved from comparison to RAST and eggNOG databases.

A dash ('-') in columns 2-4 indicates that there was no hit found and therefore no annotation term or category could be retrieved. Any other entry is the retrieved annotation term, even if it reads 'NA'. In columns 6-19 'NA' means the gene family was not found significant in that phage model.

Available at: <https://docs.google.com/spreadsheets/d/1joM5QoX5FCE3BI5vPiE3ucFwxD>
cSvRGR8XuJj82Fn6M/edit#gid=0

5) Supplementary Table S2. Probabilities of observing a given prevalence per functional category based on the cumulative density function (CDF). In the first column is noted the observed percentage of genes in a given category, as depicted in Figure 5. The second column shows the probability of observing this percentage or lower given the estimated CDF. Conversely, the third column shows the probability of observing an even higher percentage given the eCDF.

Note that although categories 'Chromatin structure and dynamics' and 'Extracellular structures' appear overrepresented in the significant gene set via the cumulative density function, this is meaningless since both of categories have been observed zero times in the significant gene set. Those two categories are overall extremely rare within our strain set which makes the cumulative density function collapse.

| **Letter** | **category** | **percent observed** | **p(CDF(x))** | **1-p(CDF(x))** | p<0.05 | direction |
| --- | --- | --- | --- | --- | --- | --- |
| 0 | No Hit | 40.1% | 0.99 | 0.01 | yes | enriched |
| B | Chromatin structure and dynamics | 0.0% | 0.98 | 0.02 | yes | enriched |
| C | Energy production and conversion | 0.6% | 0.13 | 0.87 | no |  |
| D | Cell cycle control, cell division, chromosome partitioning | 1.2% | 0.94 | 0.06 | no |  |
| E | Amino acid transport and metabolism | 1.8% | 0.06 | 0.94 | no |  |
| F | Nucleotide transport and metabolism | 0.0% | 0.08 | 0.92 | no |  |
| G | Carbohydrate transport and metabolism | 1.2% | 0.12 | 0.88 | no |  |
| H | Coenzyme transport and metabolism | 0.0% | 0.06 | 0.94 | no |  |
| I | Lipid transport and metabolism | 0.0% | 0.12 | 0.88 | no |  |
| J | Translation, ribosomal structure and biogenesis | 1.2% | 0.17 | 0.83 | no |  |
| K | Transcription | 3.0% | 0.24 | 0.76 | no |  |
| L | Replication, recombination and repair | 11.4% | 0.99 | 0.01 | yes | enriched |
| M | Cell wall/membrane/envelope biogenesis | 5.4% | 0.81 | 0.19 | no |  |
| N | Cell motility | 0.0% | 0.81 | 0.19 | no |  |
| O | Post-translational modification, protein turnover, and chaperones | 0.0% | 0.04 | 0.96 | yes | depleted |
| P | Inorganic ion transport and metabolism | 0.6% | 0.01 | 0.99 | yes | depleted |
| Q | Secondary metabolites biosynthesis, transport, and catabolism | 0.0% | 0.24 | 0.75 | no |  |
| S | Function unknown | 28.7% | 0.91 | 0.09 | no |  |
| T | Signal transduction mechanisms | 3.0% | 0.92 | 0.08 | no |  |
| U | Intracellular trafficking, secretion, and vesicular transport | 0.0% | 0.38 | 0.62 | no |  |
| V | Defense mechanisms | 1.8% | 0.33 | 0.67 | no |  |
| W | Extracellular structures | 0.0% | 0.97 | 0.03 | yes | enriched |

6) Supplementary Table S3.

List of MRSA strains included in the test set and their properties. Properties listed are as follows: Clonal complex, the lineage a strain is assigned to; isolate name; N50, a criterion of assembly quality with a high N50 being better; MLST type, the type of a strain by multi locus sequence typing (MLST) [4]; spa type, the type of a strain in an often used single locus typing system [5]; criterion for including this strain, numbers are as detailed in the list of criteria above.

Available at: https://docs.google.com/spreadsheets/d/17ciUDM7rJgmCRjMq-V_xZ23wcd2HrblF206WbtzMst0/edit#gid=0

7) Supplementary Table S4. Detailed phage typing results showing the percentage of resistant, weakly susceptible and strongly susceptible bacterial strains per phage preparation.

| phage preparation | resistant | weakly susceptable | strongly susceptable |
| --- | --- | --- | --- |
| 1N/80 | 45.9 | 22.2 | 31.9 |
| 676/F | 42.5 | 6.8 | 50.7 |
| 676/T | 30.9 | 1.0 | 68.1 |
| 676/Z | 55.6 | 3.9 | 40.6 |
| A3/R | 77.8 | 3.4 | 18.8 |
| A5/L | 51.2 | 1.4 | 47.3 |
| A5/80 | 40.1 | 4.8 | 55.1 |
| P4/6409 | 58.9 | 3.4 | 37.7 |
| phi200/6409 | 45.9 | 10.1 | 44.0 |
| MS-1 | 58.9 | 7.2 | 33.8 |
| OP_MS-1 | 54.6 | 6.8 | 38.6 |
| OP_MS-1_TOP | 50.7 | 9.7 | 39.6 |

8) Supplementary Table S5. Layout of the contingency tables used for analysis. The asterisk denotes the total sum fixed by design.

|  | *Susceptibility* |  |  |
| --- | --- | --- | --- |
| *Presence of gene family* | susceptible | resistant | Sum |
| present | a^1^ | b^2^ | a+b |
| absent | c^3^ | d^4^ | c+d |
| Sum | a+c | b+d | n* |

^1^ Number of isolates that are susceptible to the phage currently looked at and in which the current cluster is present.
^2^ Number of isolates that are resistant to the phage currently looked at and in which the current cluster is present. ^3^ Number of isolates that are susceptible to the phage currently looked at and in which the current cluster is absent. ^4^ Number of isolates that are resistant to the phage currently looked at and in which the current cluster is absent.
Both the row and column margins sum to n.

9) Details on susceptibility testing as described by Ślopek *et al* [6]

Since this reference may not be easily available to readers, we have here summarized the assessment. Ślopek *et al* describe the following 7 distinguishable lysis results ranging from negative to confluent lysis: negative, opaque lysis, < 20 plaques, > 20 plaques, 40 - 80 plaques ,semi-confluent lysis confluent lysis.

The definition of phage susceptibility we used in this analysis encompasses only the two highest lysis levels, namely confluent lysis and semi confluent lysis. We have re-run the modeling process including also the weakly sensitive levels (> 20 plaques, 40 - 80 plaques) and found no substantial difference in the modeling results. This is probably because intermediate sensitivity was rarely observed in our strain set (see Supplementary Table S4). Note that phage preparations for testing were at RTD which is a fairly potent dilution, defined as being the highest dilution that still gives confluent lysis on the designated propagating strain of *S. aureus* [7]

10) Details on Feature selection by association testing (Methods section 4.6):

We chose to employ Fischer-Boschloo’s to test for association in favor of the often used Fischer's test. This is because in contrast to the often used Fischer's, Fischer-Boschloo’s is an exact unconditional test and in total sum fixed designs unconditional test are always preferable to conditional tests for reasons detailed by Lydersen et al [8].

This first filtering step was performed inside the cross validation framework, so that the partition being tested was not included in this initial p-value based feature reduction. Due to the fact that the 2x2 tables were constructed from only the training set, some gene families in a given test set do not have a p-value associated. This specific situation arises when gene families are only present in one partition and that partition is left out of the training set. In these cases the gene family was assigned a p-value of NA (not applicable).

As can be seen in Figure 1, partition 1 is substantially larger than the other four partitions in the benchmark data, see 3.2 for further details. This potentially imposes a bias when calculating the association p-values, since these often will be driven solely by the data in partition 1. To amend that, a bootstrapping resampling procedure was applied to partition 1: When picking gene families based on a combination of partitions that includes partition 1, instead of including the full partition, a subsample of 25 strains used and was added to the other three partitions. From that data, we then created contingency tables and calculated p-values as described above. This procedure was repeated a 100 times, resulting in 100 p-values per gene family per phage interaction. We then imposed the condition that a gene family had to pass the p-value threshold of 0.01 in at least 90 of those to be selected

**References**

1. S. Monecke *et al.*, “A field guide to pandemic, epidemic and sporadic clones of methicillin-resistant Staphylococcus aureus.,” *PLoS One*, vol. 6, no. 4, p. e17936, Jan. 2011.
2. G. K. Paterson, E. M. Harrison, and M. A. Holmes, “The emergence of mecC methicillin-resistant Staphylococcus aureus,” *Trends in Microbiology*, vol. 22, no. 1. Elsevier, pp. 42–47, Jan-2014.
3. M. D. Bartels *et al.*, “Monitoring meticillin resistant Staphylococcus aureus and its spread in Copenhagen, Denmark, 2013, through routine whole genome sequencing,” *Eurosurveillance*, vol. 20, no. 17, p. 21112, Apr. 2015.
4. N. A. Saunders and A. Holmes, “Multilocus Sequence Typing (MLST) of Staphylococcus aureus,” in *Methicillin-Resistant Staphylococcus Aureus (MRSA) Protocols*, Second Edi., vol. 1085, Y. Ji, Ed. Totowa, NJ: Humana Press, 2014, pp. 113–130.
5. L. Koreen, S. V Ramaswamy, E. A. Graviss, S. Naidich, J. M. Musser, and B. N. Kreiswirth, “spa typing method for discriminating among Staphylococcus aureus isolates: implications for use of a single marker to detect genetic micro- and macrovariation.,” *J. Clin. Microbiol.*, vol. 42, no. 2, pp. 792–9, Feb. 2004.
6. S. Slopek, I. Durlakowa, A. Kucharewicz-Krukowska, T. Krzywy, A. Slopek, and B. Weber, “Phage typing of Shigella flexneri,” *Arch. Immunol. Ther. Exp.*, vol. 20, no. 1, 1972.
7. J. E. Blair and R. E. O. Williams, “Phage typing of staphylococci,” *Bull World Heal. Organ.*, vol. 24, no. 6, pp. 771–784, 1961.
8. S. Lydersen, M. W. Fagerland, and P. Laake, “Recommended tests for association in 2 x 2 tables.,” *Stat. Med.*, vol. 28, no. 7, pp. 1159–75, Mar. 2009.
