# Supplementary material for: Use of a Regression Model to Study Host-Genomic Determinants of Phage Susceptibility in MRSA
Source: Antibiotics (Basel). 2018 Jan 29;7(1):9. doi: 10.3390/antibiotics7010009 (PMC5872120; doi:10.3390/antibiotics7010009)
Supplement: Supplementary file 1 [file antibiotics-07-00009-s001.zip › Suplementary-final/table_S5.docx]

Supplementary Table S5. Layout of the contingency tables used for analysis. The asterisk denotes the total sum fixed by design.

|  | *Susceptibility* |  |  |
| --- | --- | --- | --- |
| *Presence of gene family* | susceptible | resistant | Sum |
| present | a^1^ | b^2^ | a+b |
| absent | c^3^ | d^4^ | c+d |
| Sum | a+c | b+d | n* |

^1^ Number of isolates that are susceptible to the phage currently looked at and in which the current cluster is present.
^2^ Number of isolates that are resistant to the phage currently looked at and in which the current cluster is present. ^3^ Number of isolates that are susceptible to the phage currently looked at and in which the current cluster is absent. ^4^ Number of isolates that are resistant to the phage currently looked at and in which the current cluster is absent.
Both the row and column margins sum to n.
