# Supplementary figures and images for: Use of a Regression Model to Study Host-Genomic Determinants of Phage Susceptibility in MRSA
Source: Antibiotics (Basel). 2018 Jan 29;7(1):9. doi: 10.3390/antibiotics7010009 (PMC5872120; doi:10.3390/antibiotics7010009)

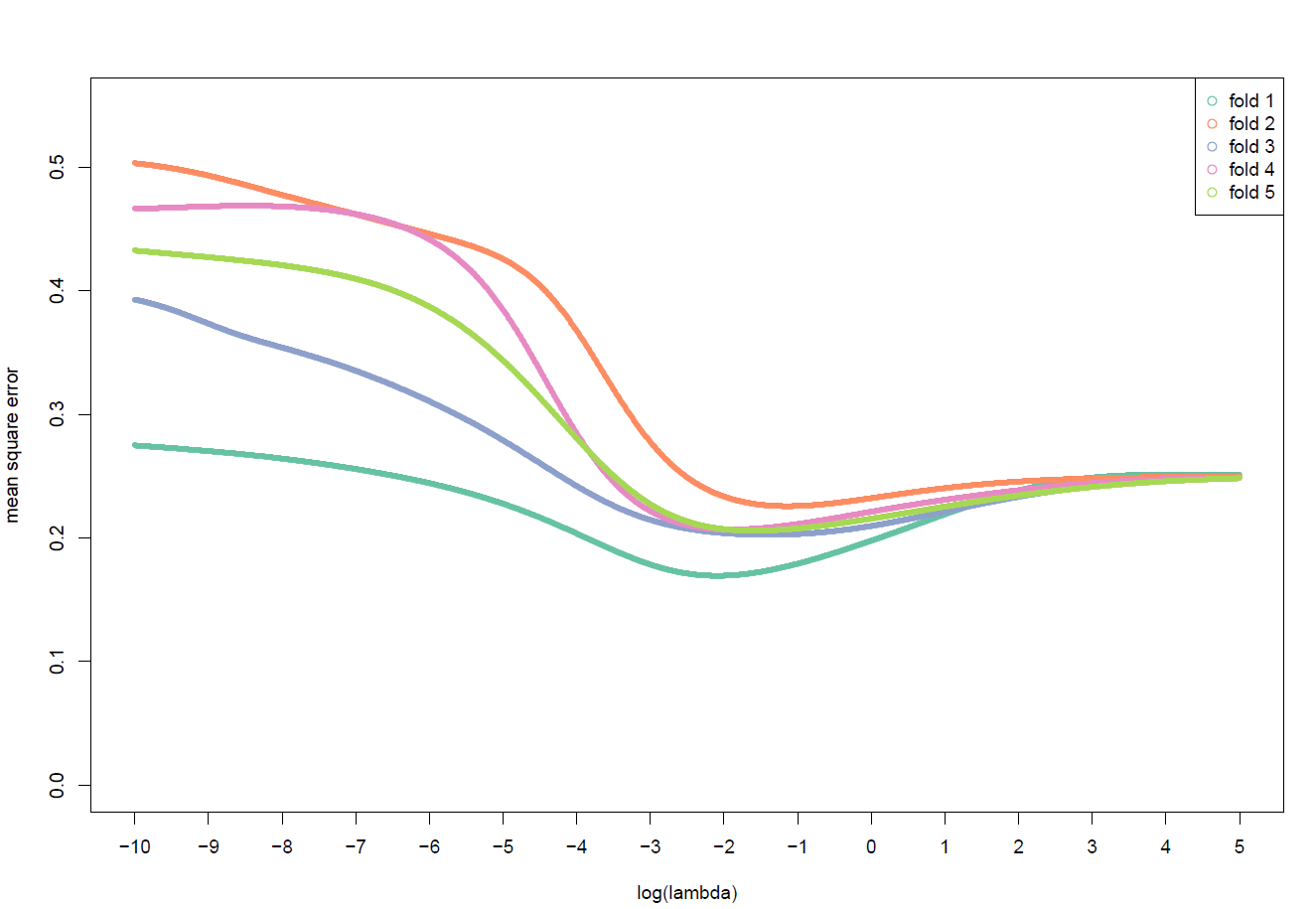

Supplement: Supplementary file 1 [file antibiotics-07-00009-s001.zip › Suplementary-final/figure_s2.PNG]
